# Closing a critical gap in perinatal mental health: The case for posttraumatic stress disorder screening in obstetric care

**DOI:** 10.1002/pmf2.70351

**Published:** 2026-07-01

**Authors:** Abigail Powers, Briana Woods‐Jaeger

**Affiliations:** ^1^ Department of Psychiatry & Behavioral Sciences Emory University School of Medicine Atlanta Georgia USA; ^2^ Department of Behavioral, Social, and Health Education Sciences Emory University School of Public Health Atlanta Georgia USA

Improving maternal mental health is a critical component of efforts to reduce the burden of maternal morbidity and mortality in the United States. Screening for perinatal depression and anxiety is widely recommended and increasingly implemented in obstetric (OB) care, as recommended by the American College of Obstetricians and Gynecologists (ACOG). These efforts represent a strong step forward in recognizing the importance of maternal mental health. Yet, posttraumatic stress disorder (PTSD) remains largely overlooked. This omission is striking given that trauma exposure is highly prevalent among women, PTSD rates in high‐risk perinatal populations can approach or exceed those of depression, and experiences in pregnancy or childbirth can be traumatic (e.g., stillbirth, serious injury). Mental health conditions are a leading cause of pregnancy‐related death in the United States [1], and trauma and stressor‐related disorders, including PTSD, are among the mental health conditions most strongly associated with adverse maternal health outcomes [2]. Yet, routine assessment for trauma and PTSD remains uncommon in OB care. As a result, trauma‐related mental health morbidity during pregnancy and postpartum commonly goes undetected [3]. There are growing calls to address PTSD in the perinatal period (e.g., [4]), but to our knowledge, no substantial changes have been made toward widespread universal screening for PTSD in this period. As perinatal mental health screening expands nationally, the continued absence of PTSD assessment represents a notable gap in maternal care. Integrating trauma‐informed PTSD screening into perinatal care represents an important step in advancing maternal mental health and improving outcomes across generations.

Trauma exposure is common across the lifespan, with most adults reporting at least one traumatic event in their lifetime. Women are two‐to‐three times more likely than men to develop PTSD following trauma exposure, increasing vulnerability for women in particular. Additionally, rates of PTSD are higher in certain groups, including those with limited socioeconomic resources and are marginalized by race or ethnicity, with estimates of current PTSD at 30% among Black pregnant women utilizing OB care from an urban safety‐net hospital [3], compared to 3.3% in general community samples of prenatal women [5]. Importantly, pregnancy represents a unique time of vulnerability for women, where both prior trauma (e.g., childhood maltreatment, interpersonal violence) and new traumatic experiences (e.g., childbirth‐related serious injury or death) during the perinatal period may contribute to PTSD.

PTSD in the perinatal period has important implications for maternal and infant health. Symptoms of PTSD, which include intrusive memories, avoidance, sleep disruption, emotional numbing, and hyperarousal, impair daily functioning. PTSD is associated with comorbid mental health symptoms and substance use, difficulties with emotion regulation, and impaired maternal functioning and well‐being. Maternal PTSD may also influence caregiving behaviors and maternal–infant bonding. Given the potential for these cascading effects, identifying PTSD during the perinatal period represents an important opportunity for early intervention.

Despite these risks, PTSD remains underdetected in perinatal care. In research conducted with pregnant Black women receiving care in an urban public hospital clinic, although a high prevalence of PTSD (∼30%) was present, PTSD diagnoses were absent from patients’ medical records [3]. Such discrepancies highlight the substantial gap between symptom burden and clinical recognition. This gap is particularly concerning in safety‐net health systems serving individuals with limited socioeconomic resources and higher exposure to structural or social stressors, where trauma exposure and mental health needs are especially high.

Several barriers contribute to the limited adoption of PTSD screening in OB settings. One is stigma about screening (e.g., concern that asking about trauma may worsen symptoms). Importantly, evidence suggests that patients generally tolerate trauma screening well and that brief trauma assessments do not increase PTSD symptoms when conducted in a supportive clinical environment [6]. In fact, many patients report appreciating the opportunity to discuss experiences that may otherwise remain unrecognized within medical care.

Another barrier is how to support patients with a positive screen when mental health resources are lacking. Many OB clinics, particularly those serving uninsured or publicly insured populations, operate within health systems with limited access to integrated behavioral health services or major strains on those systems. Clinicians may feel reluctant to identify mental health needs when resources for follow‐up care are scarce. Pregnant patients facing housing instability, food insecurity, or other social stressors may also require comprehensive support that extends beyond traditional mental health treatment. Thus, identifying local and national supports *before* screening is implemented is critical. Working within the system to build supports prior to screening implementation may be necessary and could include integrating services (e.g., individual or group therapy), creating resource packets with vetted services (local or national), and partnering with experts to train community partners to offer behavioral healthcare services through task‐shifting.

A final barrier involves the practical realities of OB care. Prenatal visits are full and have many clinical priorities, so PTSD screening can be seen as a burden within an already constrained clinical encounter. However, integrating PTSD screening into perinatal care is feasible by using streamlined workflows and brief validated screening tools [7]. One option utilized widely in primary care clinic settings is the Primary Care PTSD Screen for DSM‐5 [8], a 5‐item self‐report measure that can be added to an existing screening battery for prenatal care visits. Rather than creating a new screening infrastructure, PTSD screening could be integrated into existing mental health screening workflows already implemented in OB visits. These instruments are developed as self‐report measures and thus can be completed by the patient while waiting for their appointment or conducted with non‐specialist staff or providers (e.g., peer provider). When a positive screen occurs (determined by established cutoffs), the referral process would be flagged to offer internal or external resources already established by the OB team. Integrating PTSD screening with current mental health assessments would allow clinicians or staff to build upon established processes for administering questionnaires, reviewing results, and connecting patients with appropriate behavioral health resources. This approach aligns with broader efforts to expand integrated behavioral health models within OB settings. These tools allow clinicians to identify individuals who may benefit from direct referral and further assessment without substantially increasing clinical burden.

Screening may be particularly valuable at several key points across the perinatal period. Early pregnancy visits provide an opportunity to identify individuals with pre‐existing trauma histories or ongoing PTSD symptoms and have the greatest opportunity for early intervention before birth occurs. Later pregnancy visits may help identify patients experiencing increasing distress as childbirth approaches. Postpartum visits are also critical, as the weeks following childbirth, sometimes referred to as the “fourth trimester”, represent a period of heightened vulnerability for the emergence or exacerbation of PTSD symptoms, particularly following difficult or traumatic birth experiences [5].

Efforts to expand PTSD screening must occur within a culturally responsive trauma‐informed care (TIC) framework. TIC emphasizes safety, trust, patient autonomy, and recognition of the widespread impact of trauma. Screening should therefore be conducted in ways that respect patients’ comfort and choice, provide clear explanations for why questions are being asked, and ensure that appropriate support is available when concerns are identified. Importantly, TIC approaches also recognize the role of structural inequities in shaping trauma exposure and access to care. There may be specific needs of certain groups that must be acknowledged and addressed for PTSD screening practices to be effective. For example, minoritized groups may experience heightened medical mistrust due to negative personal experiences with medical providers or knowledge of past mistreatment of women in their community. Addressing PTSD in perinatal populations, therefore, requires attention not only to psychological symptoms but also to the broader social contexts in which perinatal patients live and their experience with systemic barriers in medical care. That may mean that PTSD screening should not look the same in every setting; more research is necessary to understand the overall impact of PTSD screening on perinatal and mental health outcomes, if it helps identify needs beyond depression and anxiety screening and expedite referrals to necessary PTSD care, and whether brief PTSD screening alone is enough for all perinatal patients. Through ongoing funded work by ourselves and others in the field, we hope to address these questions and further guide answers to not only the question of *should we screen* for PTSD, but also *how best to screen* for PTSD in varied at‐risk groups.

As perinatal care increasingly recognizes the importance of mental health, the continued absence of routine PTSD screening represents a critical gap. Trauma exposure and PTSD are common among women of reproductive age, and untreated symptoms may have intergenerational consequences. Fortunately, integrating trauma‐informed PTSD screening into perinatal care is both feasible and aligned with existing efforts to expand mental health screening in OB settings. Moving toward routine assessment of trauma and PTSD, particularly in clinical settings serving populations at elevated risk, represents an important opportunity to improve maternal mental health care and support healthier outcomes for mothers and infants.

## AUTHOR CONTRIBUTIONS

**Briana Woods‐Jaeger**: Writing—review and editing. **Abigail Powers**: Conceptualization; writing—original draft; writing—review and editing; resources.

## CONFLICT OF INTEREST STATEMENT

The authors declare no conflicts of interest.

## FUNDING INFORMATION

The authors received no specific funding for this work.

## Data Availability

Data sharing not applicable to this article as no datasets were generated or analyzed during the current study.
